# Expression of the *Streptococcus pneumoniae yoeB* Chromosomal toxin gene causes Cell Death in the model plant *Arabidopsis thaliana*

**DOI:** 10.1186/s12896-015-0138-8

**Published:** 2015-04-12

**Authors:** Fauziah Abu Bakar, Chew Chieng Yeo, Jennifer Ann Harikrishna

**Affiliations:** Centre for Research in Biotechnology for Agriculture (CEBAR) and Institute of Biological Sciences, Faculty of Science, University of Malaya, 50603 Kuala Lumpur, Malaysia; Biomedical Research Centre, Faculty of Medicine, Universiti Sultan Zainal Abidin, Medical Campus, 20400, Kuala Terengganu, Malaysia

**Keywords:** Toxin-Antitoxin system, Bacteria, *Arabidopsis thaliana*, 17-β-estradiol, Expression

## Abstract

**Background:**

Bacterial toxin-antitoxin systems usually comprise of a pair of genes encoding a stable toxin and its cognate labile antitoxin and are located in the chromosome or in plasmids of several bacterial species. Chromosomally-encoded toxin-antitoxin systems are involved in bacterial stress responses and activation of the toxins usually leads to cell death or dormancy. Overexpression of the chromosomally-encoded YoeB toxin from the *yefM*-*yoeB* toxin-antitoxin locus of the Gram-positive bacterium *Streptococcus pneumoniae* has been shown to cause cell death in *S. pneumoniae* as well as *E. coli.*

**Results:**

Induction of a YoeB-GFP fusion protein using a 17-β-estradiol-inducible plant expression system in *Arabidopsis thaliana* Col 0, was lethal in all T_2_ progeny. Examination of plants by fluorescent confocal microscopy showed GFP fluorescence in all parts of the leaves at 24 hours after 17-β-estradiol induction, continuing up to plant death. Quantitative RT-PCR analysis revealed that the expression of the *yoeB* toxin gene peaked at 3 days after induction with 17-β-estradiol, coinciding with the onset of visible effects on the plants. Moreover, we detected DNA laddering in the transgenic plants at 24 hours after *yoeB* induction, indicative of apoptosis.

**Conclusions:**

Expression of the YoeB toxin from *Streptococcus pneumoniae* is lethal in Arabidopsis. We believe this is the first report of a toxin from a bacterial toxin-antitoxin system functioning in plants. The results presented here mark an important milestone towards the development of a cell ablation based bio-containment strategy, which may be useful for functional studies and for the control of spread of transgenic plants.

## Background

Most bacteria harbour toxin-antitoxin (TA) systems, usually comprising a pair of genes coding for a toxin and its cognate antitoxin [1]. Upon antitoxin degradation, the toxin induces cell stasis or death in part of the cell population leading to the early proposal of TA systems being a form of programmed cell death in prokaryotes [2]. TA systems were originally discovered in bacterial plasmids, where they function in the maintenance of plasmids through the post-segregational killing of plasmid-free daughter cells [3-6]. Chromosomally-encoded TA systems were subsequently found to be also abundant in bacteria and archaea where they have been implicated in several cellular functions such as programmed cell death [7], stress responses [8] and in persistence and antibiotic tolerance [9]. A wide variety of TA systems have been discovered in the past decade and currently, TA systems are grouped into classes I, II, III, IV and V according to the nature and action of the antitoxin [10-13].

Type II TA systems have been most widely studied [14] and encode a toxic protein and a relatively less stable cognate proteic antitoxin. Environmental stress conditions usually lead to the induction of endogenous proteases which results in the degradation of antitoxins, thereby releasing the toxins from the inert toxin-antitoxin complex. The unbound toxin is now free to exert its lethal effect through different modes of action depending on the toxin type. The majority of characterized toxins act as endoribonucleases, some toxins interfere with DNA gyrase activity and thus function as an inhibitor of DNA replication and transcription, while other toxins inhibit translation initiation and interfere with cell wall synthesis [15,16]. The chromosomal TA systems from Gram negative bacteria are among the most prevalent and well-studied. Some of these prokaryotic toxins have been shown to have activity when expressed in eukaryotic cells such as yeasts and have been proposed to have potential application in the control of cell growth in eukaryotic cells, especially in preventing the escape of genetically modified cells [17]. The *relE* toxin of *E. coli* was demonstrated to be functional in the yeast *Saccharomyces cerevisiae* where induction of the toxin gene in transformed yeast cells inhibited growth [18]. Expression of the RelE toxin and the Kid toxin were also shown to trigger apoptosis in a human osteosarcoma cell line [18] and in HeLa cells [19], respectively. These findings eventually led to the development of a method using the Kis-Kid TA system to select for mammalian cells with a stable and high level expression of transgenes [20]. Another TA system that has been shown to be functional in eukaryotes is the Epsilon-Zeta system from plasmid pSM19035 of *Streptococcus pyogenes* where expression of the Zeta toxin in the yeast *Saccharomyces cerevisiae* was shown to be lethal [21].

Up to 10 putative TA systems have been found through a bioinformatics search of sequenced *Streptococcus pneumoniae* genomes [22]. Out of these, three have been demonstrated to be functional *bona fide* TA systems, namely RelBE2 [23,24], YefM-YoeB_Spn_ [22,25] and PezAT [26]. The YoeB toxin homologues have been shown to be endoribonucleases and overexpression of the YoeB_Spn_ toxin led to cell death in both *S. pneumoniae* and *E. coli* [25].

To our knowledge, there have been no reports on the functionality of bacterial TA systems in plants. Here, we investigate the effects of expressing the *yoeB*_*Spn*_ toxin gene in *Arabidopsis thaliana* as a model plant system using a 17-β-estradiol-inducible expression system. Ultimately, this work may lead to the development of a bio-containment strategy, which may be useful for preventing the release of unwanted genetically modified plants to the environment, for the development of male sterile plants for hybrid seed production as well as the development of a novel cell ablation system for functional studies in plants.

## Results

### Analysis of transgenic plants

**Figure 1:**
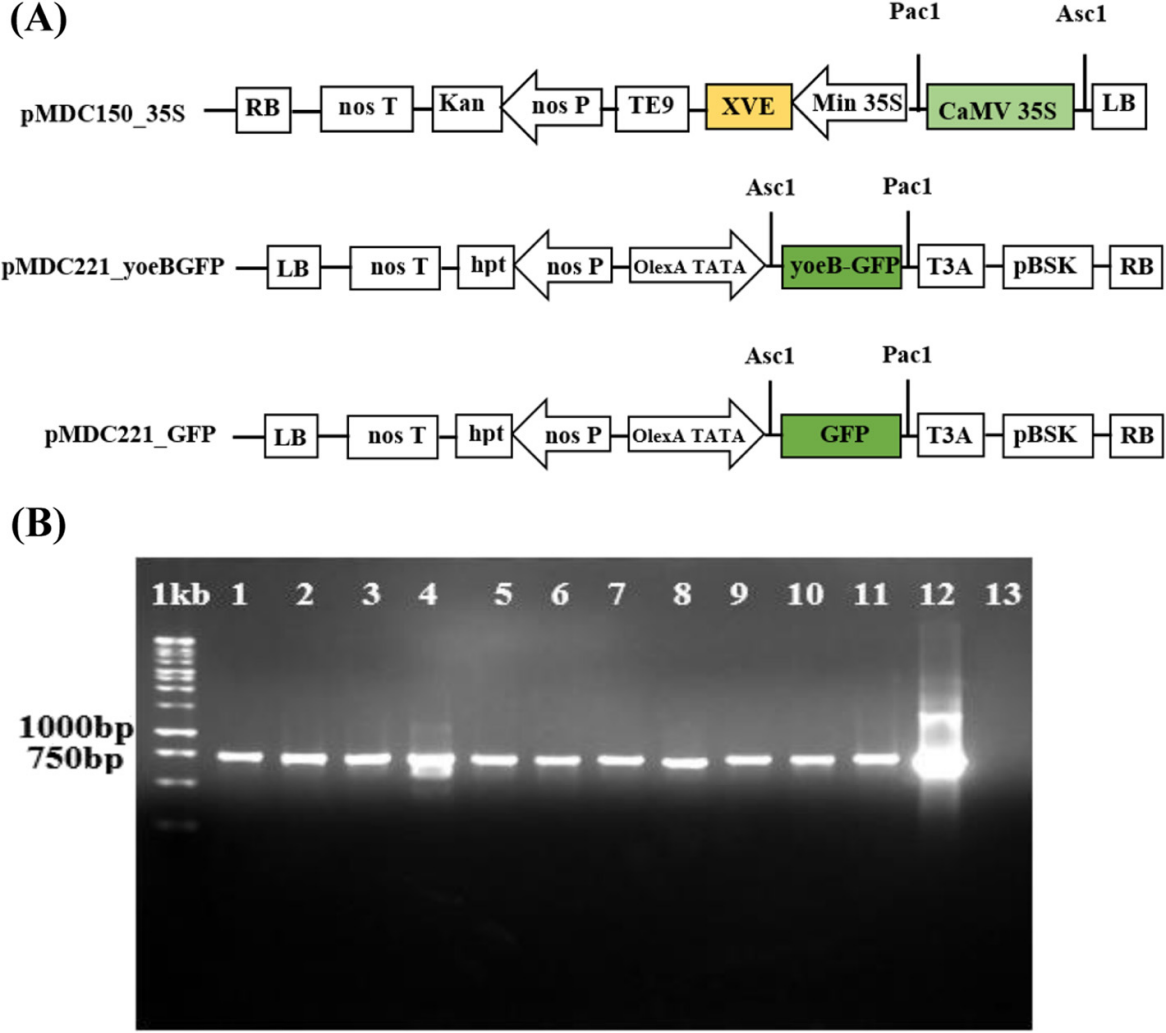
Vector constructs and PCR amplification from four-week-old T_2_ transgenic *A. thaliana* using *GFP*-specific primers. (**A**) Schematic diagram of the plant inducible expression vectors that were constructed for this study based on [27]. (**B**) Agarose gel electrophoresis of PCR products using *GFP*-specific primers; samples were total DNA from plants transformed with pMDC221_yoeBGFP (lanes 1–3: transgenic line 1; lanes 4–5: transgenic line 2; lanes 6–8: transgenic line 3; lanes 9–11: transgenic line 4) or with pMDC221-GFP (lane 12) and showing the expected amplified product of 732 bp; Lane 13: sample was total DNA from a wild type *Arabidopsis* plant (negative control); 1 kb: 1 kb DNA ladder Marker (Fermentas).

A 17-β-estradiol-inducible two-component system [27] was used to obtain transgenic *Arabidopsis thaliana* for controlled expression of the *yoeB*_*Spn*_ toxin gene, cloned as a translational fusion with the *GFP* gene in the responder vector pMDC221_yoeBGFP while the CaMV 35S promoter was cloned into the activator vector pMDC150_35S to drive the constitutive expression of the 17-β-estradiol-responsive *XVE* transcriptional activator (Figure 1A).

**Figure 2:**
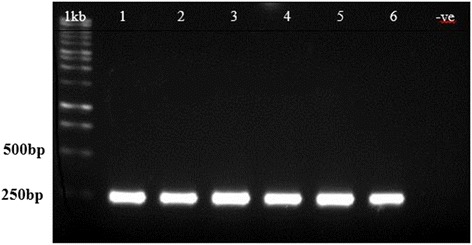
PCR confirmation of *yoeB*_Spn_ toxin gene from four-week-old-T_2_ transgenic *A. thaliana* using *yoeB*-specific primers. Samples were total DNA extracted from the T_2_ transgenic plants showing the expected amplified product of 255 bp. Lanes 1–2: transgenic line 1; lanes 3–4: transgenic line 2; lanes 5–6: transgenic line 3; lane -ve: DNA from a wild type *Arabidopsis* plant as negative control; 1 kb: 1 kb DNA ladder Marker (Fermentas).

**Figure 3:**
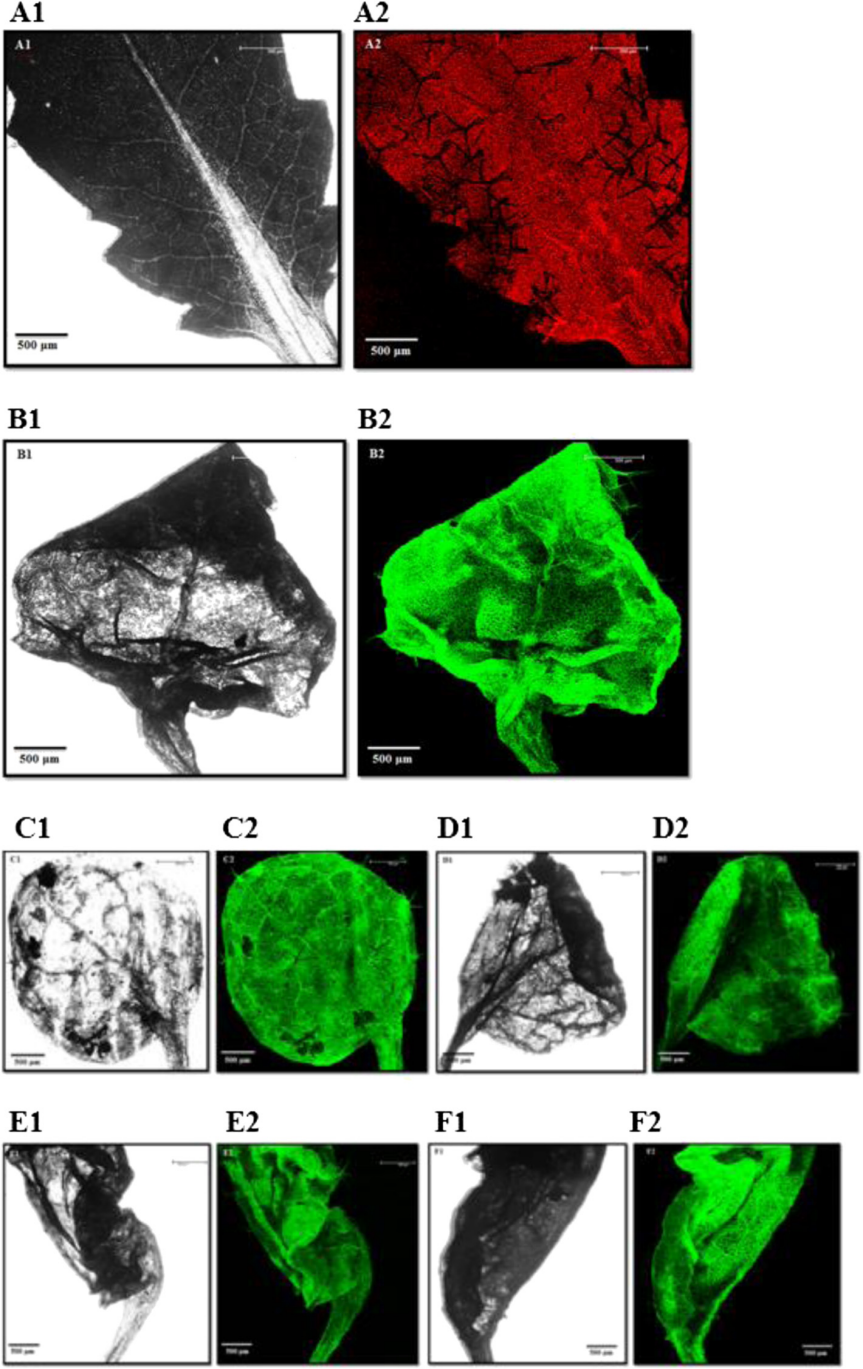
GFP fluorescence image of rosette leaves of T_2_ *Arabidopsis thaliana.* Plants transformed with pMDC221_yoeBGFP were induced with 100 μM 17-β-estradiol at 4 weeks after selection. (**A**) no induction; (**B**) 1 day after induction; (**C**) 3 days after induction; (**D**) 6 days after induction; (**E**) 8 days after induction; and (**F**) 9 days after induction. 1 and 2 represent bright field and fluorescent images, respectively. Scale bars = 500 μm.

Five independent transformation experiments were conducted and 4 independent transgenic lines were selected for further analysis. Out of 100 T_2_ YoeB_Spn_-GFP transgenic plants that grew on the selection medium, 11 were randomly chosen for genomic DNA preparation and PCR amplification using *GFP*-specific primers (Figure 1B, lanes 1 – 11). The presence of a 732 bp band indicated the presence of the *yoeB*_*Spn*_*-GFP* fusion transgene and, thus, the successful integration of the transgene into the *Arabidopsis* plants. This band was also present in the positive control plants expressing *GFP* alone from vector pMDC221_GFP (Figure 1B, lane 12) and was absent in the negative control (wild type) plant (Figure 1B, lane 13). Similarly, when using *yoeB*_*Spn*_-specific primers, the presence of a *yoeB*_*Spn*_-specific band was also observed in the transgenic plants (Figure 2). When the leaves of the transgenic plants were examined under fluorescence confocal microscope, GFP fluorescence was detectable in all parts of the leaves 24 hours after 17-β-estradiol induction (Figure 3). Leaves showed GFP fluorescence at days 3 and 6 after induction, and even at days 8 and 9, after plant death (Figure 3). Distortion of the newly emerging rosette leaves and necrotic symptoms in all leaves were observed from day 3 onwards, as shown in Figure 3.

### Effects of *yoeB*_*Spn*_-*GFP* expression on the transgenic *Arabidopsis thaliana* after induction with 17-β-estradiol

**Figure 4:**
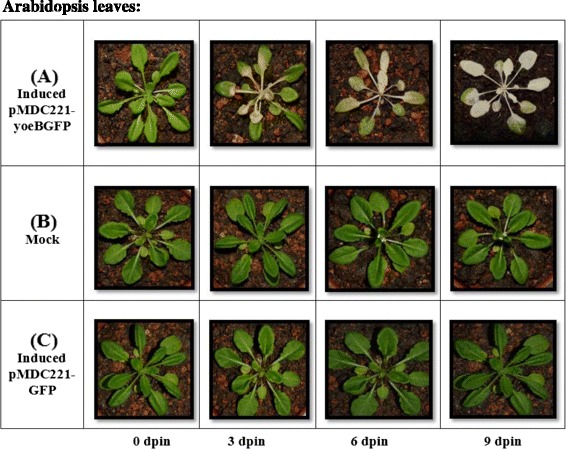
Effects of *yoeB*_Spn_-*GFP* expression on *Arabidopsis thaliana.* The appearance of transgenic T_2_ *A. thaliana* over a time course of 9 days. (**A**) 17-β-estradiol induced plants with pMDC221_yoeBGFP, (**B**) mock induced (with ethanol) plants with pMDC221_yoeBGFP and (**C**) 17-β-estradiol induced pMDC221_GFP.

Induction of expression of the *yoeB*_*Spn*_ toxin in four-week-old T_2_ generation transgenic *A. thaliana* with 17-β-estradiol, resulted in plant defects and tissue necrosis by 3 days after induction, followed by plant death over a period of 9 days (Figure 4). The transgenic plants showed severe discolouration over time and were completely discoloured on the 9^th^ day after induction (Figure 4A, 9dpin), whereas no such abnormalities were observed in the control (mock induced) transgenic *A. thaliana* that were sprayed with ethanol instead of 17-β-estradiol. Similar results were observed for all four transformed plant lines. The control plants grew normally and were able to produce flowers (Figure 4B, 9 dpin). Likewise, transgenic plants that expressed only *GFP* did not show abnormalities following 17-β-estradiol induction (Figure 4C), indicating that the plant death was due to the expression of the *yoeB*_*Spn*_ toxin.

**Figure 5:**
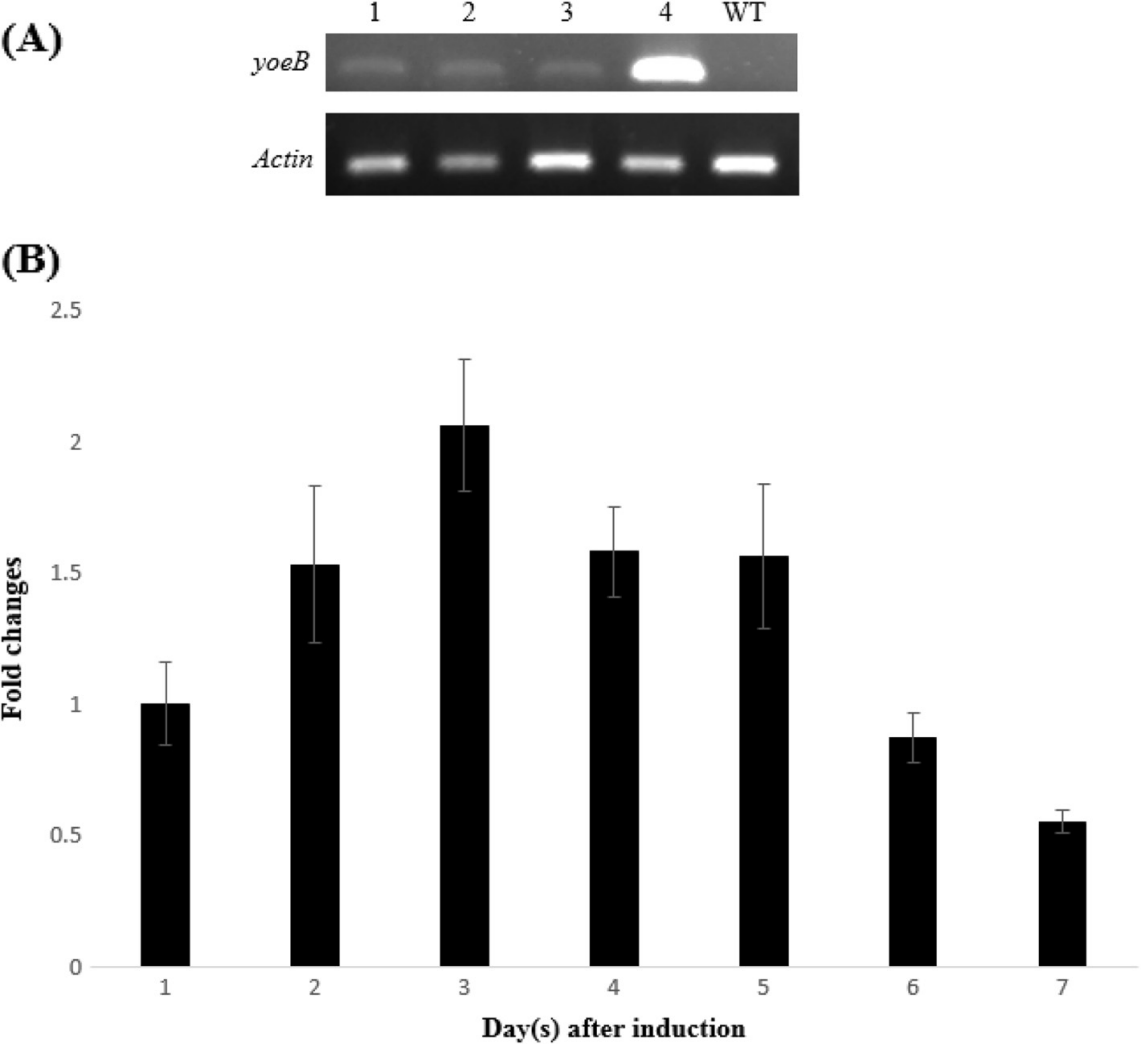
Transcript analysis of T_2_ transgenic *A. thaliana* after induction with 17-β-estradiol. (**A**) Agarose gel following RT-PCR with *yoeB* toxin and the control *Actin* primers from four different plants transformed with pMDC221_yoeBGFP and wild type control 24 hours after induction; (**B**) expression levels of the *yoeB* toxin in *A. thaliana* plants transformed with pMDC221_yoeBGFP from day 1 – day 7 after induction as determined by qRT-PCR. Each bar represents the mean and standard error for 3 biological replicates which were chosen randomly from 100 T_2_ transgenic plants from line 1.

In RT-PCR analysis of transgenic plants, the *GFP* and *Actin* transcripts were detected by the presence of amplicons of the expected sizes of 168 bp and 100 bp, respectively (Figure 5A). Quantitative RT-PCR analysis revealed that the expression of the *yoeB*_*Spn*_ toxin gene in transgenic *Arabidopsis* increased up to 3 days following induction with 17-β-estradiol, after which it decreased (Figure 5B).

### Expression of the YoeB_Spn_ toxin triggers DNA laddering in *A. thaliana*

**Figure 6:**
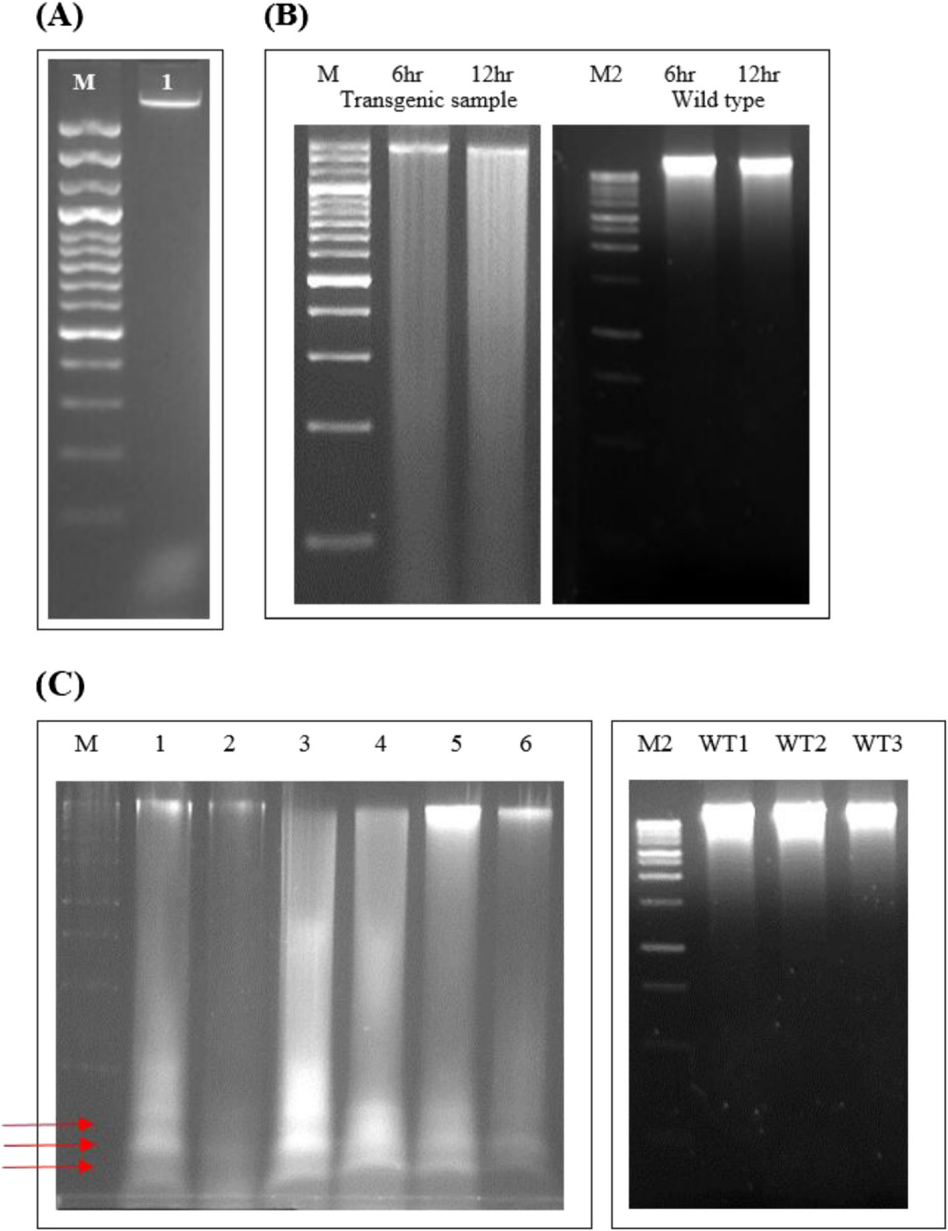
Laddering of nuclear DNA from transgenic *A. thaliana.* DNA extracted from *A. thaliana* and electrophoresed for 3 h on 1.8% agarose. (**A**) Lane 1: DNA from non-induced plant transformed with pMDC221_yoeBGFP. (**B**) DNA isolated from plants transformed with pMDC221_yoeBGFP (left) and wild type plants (right) at 6 and 12 hours after induction. (**C**) DNA isolated from plants 24 hours after induction with 17-β-estradiol; lanes 1–6: plants transformed with pMDC221_yoeBGFP; Lanes WT1-WT3: wild type. M: 100 bp DNA ladder (Fermentas, USA); M2:1 kb DNA ladder (Fermentas, USA). Arrows indicate DNA laddering or fragmentation suggestive of apoptosis.

The results so far indicated that expression of the YoeB_Spn_ toxin is lethal in *Arabidopsis*. To investigate the possibility that the lethality of YoeB_Spn_ is due to activation of apoptosis, a DNA fragmentation assay was carried out on 17-β-estradiol-induced *Arabidopsis* samples. Genomic DNA was extracted from 6 randomly selected transgenic plants at 6 h, 12 h and 24 h after induction. Agarose gel electrophoresis of the extracted genomic DNA indicated that DNA fragmentation was evident at 24 h after induction where distinct oligonucleosomal DNA fragments ranging from 180 – 200 bp were observed in all the sampled plants (Figure 6C), suggestive of apoptosis. These oligonucleosomal fragments were not evident in extracted genomic DNA 6 h and 12 h after induction but smearing of DNA was observed (Figure 6B). Such fragmentation was also not observed in the non-induced transgenic plant where the genomic DNA isolated formed a single high molecular weight band following gel electrophoresis (Figure 6A).

## Discussion

To date, three chromosomally-encoded TA systems from *Streptococcus pneumoniae* have been studied in detail, namely *relBE*-2 [23,24], *pezAT* [26] and *yefM-yoeB* [22,25]. Overproduction of YoeB_Spn_ was shown to be toxic to *S. pneumoniae* and *E. coli* cells, inhibiting cell growth and reducing cell viability [25]. Here we report the cloning and expression of *yoeB*_*Spn*_ from *Streptococcus pneumoniae* as a *yoeB*_*Spn*_-*GFP* fusion transgene in the model plant *Arabidopsis thaliana*. As it was possible that expression of the *yoeB*_*Spn*_ toxin gene would be lethal to the plant, we placed the *yoeB*_*Spn*_ toxin gene construct under the control of a strictly inducible promoter. Thus, a 17-β-estradiol-inducible expression system was used in this study to observe the effects of *yoeB*_*Spn*_ toxin gene expression in *A. thaliana*.

*A. thaliana* transformed with pMDC221-yoeBGFP was not detectably different from the wild type in the absence of 17-β-estradiol, however, after induction, plant leaves were distorted and had severe lesions (Figures 3 and 4). Quantitative RT-PCR analysis confirmed that the *yoeB*_*Spn*_*-GFP* fusion gene was expressed from day 1 after induction (Figure 5B) and RNA sampling stopped at day 7 because all the plants were dead by day 8 (indicated by severe discoloration of the rosette leaves in Figure 4A, 9dpin). The presence of fluorescence showed that the *yoeB*_*Spn*_*-GFP* gene was functionally expressed in the transgenic *A. thaliana* and that the YoeB_Spn_-GFP protein is stable in transgenic *A. thaliana* as the fluorescence was observed even at days 8 and 9, after plant death (Figure 3). Expression of the YoeB_Spn_-GFP protein was clearly detrimental to the transgenic plants producing distorted leaves and leading to 100% death of the T_2_ generation transgenic plants by the 8^th^ day after induction with 17-β-estradiol. As the plants with induced expression of only GFP, from the control vector pMDC221_GFP, did not show any leaf distortion (Figure 4C), we conclude that the symptoms and DNA damage seen in plants expressing the YoeB_Spn_-GFP fusion are due to expression of the toxin. Few studies have investigated the effects of the expression of bacterial TA systems in eukaryotic cells. One of the earliest studies of such heterologous expression reported the toxic effects of the *E. coli*-encoded RelE toxin in yeast cells [17]. These findings led to the proposal that *relE* could be used to restrict growth of genetically modified yeast strains to controlled environments, i.e., as a biocontainment mechanism to prevent the accidental escape of genetically modified yeasts [17,28]. The RelE toxin functions as an inhibitor of translation in its native *E. coli* host and also when expressed in *Streptococcus pneumoniae* [18]. RelE was subsequently shown to induce apoptosis in the human osteosarcoma cell line U2OS where characteristic DNA laddering was observed besides morphological changes such as membrane blebbing and chromatin condensation [18]. Since the YoeB toxin comes from the RelE superfamily of toxins [1,14], and also functions as an inhibitor of translation in *E. coli* [29], it was expected that this toxin would also be functional in eukaryotic cells and thus may lead to apoptosis when expressed in *Arabidopsis*. Similarities have been shown to exist between programmed cell death in plants and apoptosis in animals [30], and DNA laddering associated with programmed plant cell death has been reported [31-33]. The uniform degradation of genomic DNA into oligomers of apporoximately 180–200 bp, or multiples of that, characterizes internucleosomal cleavage of DNA. Such fragmentation is a biochemical hallmark of apoptosis and was originally described in animal cells [34]. We detected the oligonucleosomal DNA ladder in YoeB_Spn_-GFP-expressing plants 24 h after induction, suggesting that expression of YoeB_Spn_ may have triggered apoptosis, possibly mimicking mechanisms that are common to both animal and plant cell death programs. Besides RelE, the Kid toxin from the Kis-Kid TAS has been shown to be functional in several eukaryotic cells by inhibiting proliferation in yeast, the frog *Xenopus laevis* and human cell lines [19]. Kid was demonstrated to trigger apoptosis in HeLa cells with widespread cell death occuring beyond three days of Kid expression and total cell death after 15 days [19]. Thus, although the oligonucleosomal DNA laderring observed in the YoeB_Spn_-GFP-expressed *Arabidopsis* is suggestive of apoptotic cell death, previous results from the RelE [18] and Kid [19] studies are indicative of the bacterial toxin triggering apoptosis.

It was reported that the bacterial YoeB_Spn_ toxin caused cell death in both *S. pneumoniae* and *E. coli* but this toxin can be neutralized by tight binding with the cognate YefM_Spn_ antitoxin [25]. This indicates that regulated expression of *yoeB*_*Spn*_ and *yefM*_*Spn*_ might be used to kill particular cells in a selective way. This could be achieved by expressing these two genes under the control of promoters that are, respectively, induced and repressed in these cells, and that have the inverse behaviour in normal cells. Furthermore, RelE and YoeB have been shown to have similar folding as the RNase Barnase [35], a protein with a characteristic microbial RNase fold which has been used in plant ablation studies [36]. Previously, *Bacillus amyloliquefaciens* Barnase and Barstar genes were used to genetically engineer a new system of male fertility control in higher plants [36,37]. Barstar binds specifically with Barnase, forming highly stable complexes that could inhibit Barnase from functioning [38,39]. We showed that the expression of YoeB_Spn_ toxin is lethal to *Arabidopsis thaliana* and this could form the platform of an inducible plant cell ablation system where it would have potential application in biotechnology such as tissue specific expression to ablate pollen formation for the development of male sterile plants for containment of transgenic plants or for hybrid seed production. The conditional expression of the *yoeB*_*spn*_ toxin gene could be used to contain unwanted genetically-modified plants arising from accidental out-crossing events, without danger to unmodified plants. Under this condition, the presence of inducer would convert an inert toxin to an active toxin that kills the transgenic plants. Future studies can be carried out to see whether the expressed toxin can be neutralized by its cognate antitoxin. Experiments analysing the effects of co-expression of the *yefM-yoeB*_*Spn*_ toxin-antitoxin gene system in *Arabidopsis thaliana* are in progress.

## Conclusions

This study shows that the *S. pneumoniae*-encoded *yoeB*_*Spn*_ toxin is functional and lethal in *Arabidopsis* plants and that the gene can remain in the transgenic plant genome without any adverse effects until its expression is induced by the inducer. The results presented here mark an important milestone towards the development of a bio-containment strategy, which may be useful for preventing the release of unwanted genetically modified plants to the environment, for the development of male sterile plants for hybrid seed production as well as the development of a novel cell ablation system for functional studies in plants.

## Methods

### Ethics

This research did not involve any human subjects (including human material or human data), or animals or endangered or protected plant species as materials.

### Gene isolation, plasmid construction and *Agrobacterium* transformation

The *yoeB*_*Spn*_ toxin coding sequence (GI 15903627) from *Streptococcus pneumoniae* was amplified as a 255 bp fragment by PCR from the construct, pET28a_HisYefMYoeB [22] with primers yoeB_F: 5’-CACCATGCTACTCAAGTTTA-3’ and yoeB_R: 5’-*GGATCC*GTAATGATCTTTAAA-3’. The *Bam*HI restriction site was included at the 5’ end of the *yoeB*_*Spn*_ reverse primer and the 5’ end of the *GFP* forward primer (indicated in italics) to facilitate construction of the fusion product. Following digestion with *Bam*HI, the *yoeB*_*Spn*_ amplified product was ligated to a *Bam*HI-digested GFP coding sequence that was amplified as a 732 bp fragment from pCAMBIA 1304 (CAMBIA Co. Australia) with primers GFP_F: 5’-*GGATCC*ATGGTAGATCTGA-3’ and GFP_R: 5’-TTAAGCTTTGTATAGTTCAT-3’. The CaMV35S promoter was also amplified from pCAMBIA 1304 with primers 35S_F: 5’-CACCGCGTATTGGCTAGAGCAG-3’ and 35S_R: 5’-AGAGATAGATTTGTAGAGAGAGACTGG-3’. PCR conditions for all amplification reactions were: 95°C for 2 min, followed by 30 cycles of 1 min at 95°C, 1 min at 51-57°C, 1 min at 72°C and final extension of 4 min at 72°C. After ligation, the 987 bp *yoeB*_*Spn*_*-GFP* fusion construct and the 800 bp CaMV 35S promoter were separately cloned into the Gateway pENTR_D_TOPO cloning vector (Invitrogen, USA) according to the manufacturer’s instructions. The presence of inserts in selected *E. coli* Top10 transformants were confirmed by colony PCR using the following primers: M13_F: 5’- GTAAAACGACGGCCAGT-3’ and GFP_R primer as above which resulted in an amplicon of approximately 1187 bp for the *yoeB*_*Spn*_*-GFP* construct, andM13_F: 5’- GTAAAACGACGGCCAGT-3’ and 35S_R: 5’-AGAGATAGATTTGTAGAGAGAGACTGG-3’ primers which resulted in an amplicon of approximately 950 bp for the CaMV 35S construct. All the Gateway entry clones obtained were validated by conventional Sanger DNA sequencing before cloning into Gateway destination vectors. The plant inducible expression vectors, pMDC150 and pMDC221 were developed to enable non-leaky conditional gene expression in selected plant tissues or cell types [27]. The *yoeB*_*Spn*_*-GFP* and CaMV 35S promoter fragments were transferred into Gateway pMDC221 and pMDC150 respectively using LR clonase reactions (Invitrogen, USA). The constructs were transformed into *E. coli* Top10 and transformants were checked by colony PCR analysis using gene specific primers. Resulting recombinants were designated pMDC221_yoeBGFP and pMDC150_35S after verifying the constructs by sequencing. The positive control, pMDC221_GFP (i.e., pMDC221 harbouring just the *GFP* coding sequence alone) was also generated using the same methods as above. Each recombinant construct was separately transformed into *Agrobacterium tumefaciens* strain LBA 4404 using a freeze and thaw method [40] and transformed colonies confirmed by PCR amplification of 800 and 987 bp bands for CaMV35S (using primers 35S_F and 35S_R) and *yoeB*_*Spn*_*-GFP* (using primers yoeB_ F and GFP_R), respectively.

### Plant material, growth conditions and plant transformations

*Arabidopsis thaliana* Col 0 was grown with a 16 h photoperiod at 22°C until the bolting stage. *Agrobacterium*–mediated transformation with both recombinant constructs, pMDC150_ 35S and pMDC221_*yoeBGFP*, was carried out using a double floral dip method [41]. Positive control pMDC221_GFP was also transformed into the plant together with pMDC150_35S using the same method. Five independent transformation experiments were conducted and four lines were chosen for phenotypic evaluation (observation of plant death) and PCR analysis.

### Selection of transgenic *A. thaliana*

T_0_ seeds harvested from transformed plants were selected on dry silicon dioxide sand (Fluka) containing ¼ strength MSO solution in the presence of 50 μg/ml Hygromycin and 50 μg/ml Kanamycin [42] to generate T_1_ plantlets. Two-week-old T_1_ plants were transferred into soil and grown in a BSL2 greenhouse until mature and the seeds were harvested for T_2_ selection. T_2_ transgenic plants were selected using the same selection method and positive transformants were used in further analysis.

### PCR analysis of transgenic plants

Genomic DNA was extracted from fresh leaf tissues of four-week-old plants using a CTAB method [43]. Presence of the transgene was confirmed with PCR using the GFP-F and GFP-R primers previously used for cloning*. yoeB*-specific primers were also used to confirm the presence of the *yoeB*_*Spn*_*-GFP* transgene. PCR conditions for all amplification reactions were: 95°C for 2 min, followed by 30 cycles of 30 s at 95°C, 30 s at 57°C, 2 min at 72°C and final extension of 4 min at 72°C.

### *yoeB*_*Spn*_*-GFP* gene induction in transgenic plants

Four-week-old transgenic *A. thaliana* plants were induced with 100 μM 17-β-estradiol in the presence of 0.02% Tween-20 [44] using an artist paint brush [27]. The plants were covered with plastic overnight to ensure high humidity. The rosette leaves were harvested every 24 hours after induction for 7 days and stored at −80°C prior to RNA extraction. Phenotypes of the transgenic and control plants were monitored daily for 2 weeks to observe the effects of the expression of the *yoeB*_*Spn*_ toxin gene. GFP expression was monitored in plant samples on day 1, 3, 6, 8 and 9 after induction and observed by confocal microscopy under a Leica DMIRE2 microscope equipped with a Leica TCS SP5 II laser scanning device.

### Quantitative real-time reverse transcriptase-PCR (qRT-PCR)

Total RNA was extracted from *A. thaliana* using an RNeasy Plant Mini Kit (Qiagen, Germany) and then treated with RNase-free DNase I Amplification Grade (Invitrogen, USA) according to the manufacturer’s instructions. cDNA was synthesized from 1 μg of RNA in two steps using a High-Capacity cDNA Reverse Transcription Kit (Applied Biosystem, USA) under the following conditions: 25°C for 10 minutes, 37°C for 120 minutes and ending with 85°C for 5 minutes.

Gene expression was analysed using an ABI 7000 sequence detection system according to the manufacturer’s protocol (Applied Biosystems, USA). Each reaction consisted of 1 μM of both forward and reverse primers, 100 ng of cDNA as template, and 1× SYBR Green Master mix (Applied Biosystem, USA) in a final volume of 25 μl. The following primers were used for amplifying the cDNA: qPCR_161_GFPF: 5’-GGACGACGGGAACTACAAGA-3’ and qPCR_161_GFPR: 5’-CGGCCATGATGTATACGTTG-3’. The reaction settings consisted of an initial denaturation step of 5 min at 94°C followed by 40 cycles of 10 s at 94°C, 15 s at 60°C, and 15 s at 72°C. The *A. thaliana* actin gene, which was amplified using the Actin_forward primer: 5’- CCAGTGGTCGTACAACCGGTAT – 3’ and Actin_reverse primer: 5’ – ACCCTCGTAGATTGGCACAGT – 3’ was used as the reference to normalize gene expression across the samples [45]. Fluorescence readings were taken at the end of each cycle and the specificity of amplification as well as the absence of primer dimers was confirmed with a melting curve analysis at the end of each reaction. Fluorescence and Ct values were exported and analysed in MS Excel (Microsoft, USA). The day 1 sample was set as 1.0 and used as the calibrator.

### Apoptosis DNA ladder fragmentation assay

Genomic DNA was extracted from plant tissues 6, 12 and 24 hours after induction with 17-β-estradiol using an Apoptotic-Ladder Kit (bioPLUS, USA) according to the manufacturer’s instructions. Extracted DNA samples were resuspended in TE buffer and were electrophoretically separated on a 1.8% agarose gel at 60 V for 2 hours.

## Abbreviations

CTAB: Cetyltrimethylammonium bromide
GFP: Green fluorescent protein
*Spn*: *Streptococcus pneumoniae*
TA: Toxin-antitoxin
