# Erratum to: Expression of the *Streptococcus pneumoniae* yoeB Chromosomal toxin gene causes Cell Death in the model plant *Arabidopsis thaliana*

**DOI:** 10.1186/s12896-015-0220-2

**Published:** 2015-10-29

**Authors:** Fauziah Abu Bakar, Chew Chieng Yeo, Jennifer Ann Harikrishna

**Affiliations:** Centre for Research in Biotechnology for Agriculture (CEBAR) and Institute of Biological Sciences, Faculty of Science, University of Malaya, 50603 Kuala Lumpur, Malaysia; Biomedical Research Centre, Faculty of Medicine, Universiti Sultan Zainal Abidin, Medical Campus, 20400, Kuala Terengganu, Malaysia

Following publication of our paper in BMC Biotechnology [1] we discovered that some of the images used in Figure four (Fig. 1 here) were duplicates. Because of this observation, we have made corrections to replace the duplicated images with the correct original images for Figure four B (6 dpin), Figure four C (6 dpin) and Figure four C (9dpin).

**Fig. 1:**
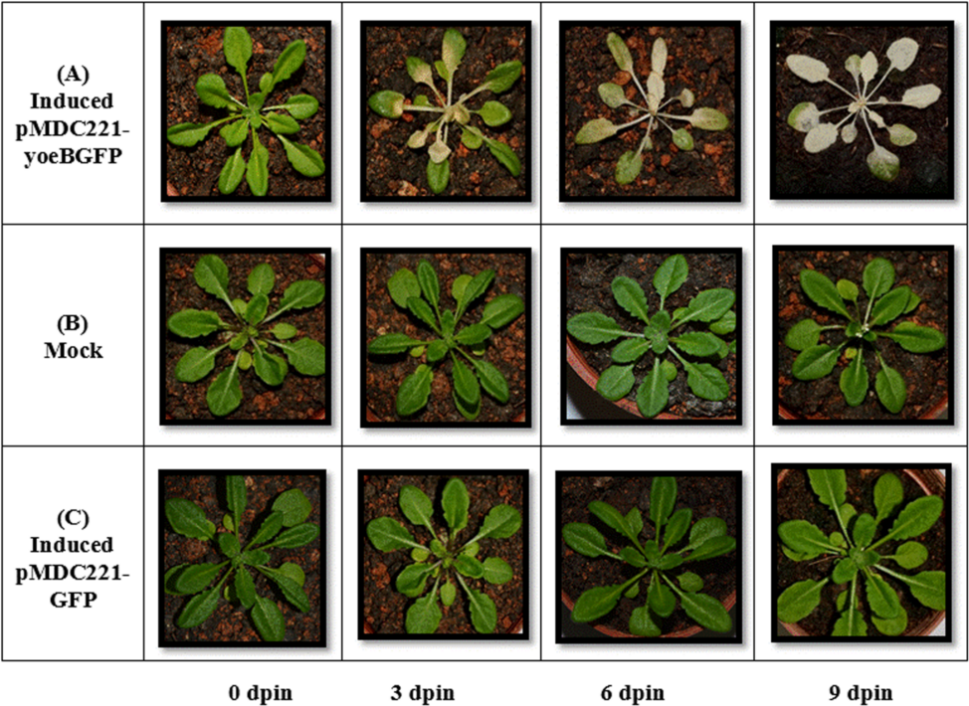
Effects of yoeBSpn-GFP expression on *Arabidopsis thaliana*. The appearance of transgenic T2 *A. thaliana* over a time course of 9 days. **a** 17-β-estradiol induced plants with pMDC221_yoeBGFP, **b** mock induced (with ethanol) plants with pMDC221_yoeBGFP and **c** 17-β-estradiol induced pMDC221_GFP
